# *ZmHMA3*, a Member of the Heavy-Metal-Transporting ATPase Family, Regulates Cd and Zn Tolerance in Maize

**DOI:** 10.3390/ijms241713496

**Published:** 2023-08-30

**Authors:** Changjian Liao, Youqiang Li, Xiaohong Wu, Wenmei Wu, Yang Zhang, Penglin Zhan, Xin Meng, Gaojiao Hu, Shiqi Yang, Haijian Lin

**Affiliations:** 1 Technical Research Center of Dry Crop Variety Breeding in Fujian Province, Crop Research Institute, Fujian Academy of Agricultural Sciences, Fuzhou 350013, China; liaocj1978@163.com (C.L.);; 2 State Key Laboratory of Crop Gene Exploration and Utilization in Southwest China, Maize Research Institute, Sichuan Agricultural University, Chengdu 611130, China; youqiangli2020@163.com (Y.L.);

**Keywords:** maize, cadmium, zinc, *HMA3*, functional analysis

## Abstract

The pollution of heavy metals is extremely serious in China, including zinc (Zn), copper (Cu), lead (Pb), and cadmium (Cd). Heavy-metal-transporting ATPase (HMA) belongs to a subfamily of the P-ATPase family, which absorbs and transports Zn, Cu, Pb, and Cd in plants. Here, we describe a *ZmHMA*-encoding HMA family protein that positively regulates Cd and Zn tolerance. The real-time fluorescence quantification (RT-PCR) results revealed that *ZmHMA3* had a high expression in B73, and the expression of *ZmHMA3* was sensitive to Cd in yeast cells, which was related to Cd accumulation in yeast. Additionally, the *Arabidopsis thaliana* homologous mutants of *AtHMA2* showed Cd sensitivity compared with WT. The overexpressing *ZmHMA3* plants showed higher tolerance under Cd and Zn stresses than the wild type. The overexpression of *ZmHMA3* led to higher Cd and Zn accumulation in tissues based on the subcellular distribution analysis. We propose that *ZmHMA3* improves maize tolerance to Cd and Zn stresses by absorbing and transporting Cd and Zn ions. This study elucidates the gene function of the *ZmHMA3* response to Cd and Zn stress and provides a reference for improving the characteristics of heavy metals enrichment in existing maize varieties and the plant remediation technology of heavy-metal-contaminated soil.

## 1. Introduction

Due to environmental degradation and industrial wastewater discharge, soil contains excessive heavy metals in China [1]. Soil with high levels of Cd and Zn will be harmful to human health and destroy the ecological environment [2,3,4]. Cd is a nonessential element in the process of plant growth and development [5]. Excessive Cd has severe adverse effects on plant growth, such as leaf chlorosis, plant wilting, and growth inhibition, in addition to negatively affecting protein activity, cell membrane structure, and photosynthetic and respiratory systems [6]. Zn is an important component associated with enzyme synthesis in plants. However, excessive Zn leads to root tip damage, leaf yellowing, and dwarfing [7]. Maize, as one of the world’s major food crops, has a global planting area of up to 118 million hectares and a total output of approximately 717 million tons [8,9,10,11,12,13]. Due to maize’s high biological yield, it can be used as a model plant for repairing heavy metal pollution in soil compared with wheat and rice. Interestingly, a previous investigation of maize tolerance revealed no adverse reactions in some mildly Cd-contaminated soils [14]. Unfortunately, when Cd content increases, resulting in maize yield reduction, the absorption of other essential elements such as Ca, Zn, and Fe is also affected [15]. However, studies on Zn pollution are focused on heavy metals such as Cd, Zn, As, and Pb in soils and the effects of these compounds on maize [16,17].

There are five subfamilies of P-type ATPase: heavy metal ATPase (P1B), Ca^2+^-ATPase (P2A and P2B), H^+^-ATPase (P3A), aminophospholipid ATPase (P4), and a branch with unknown specificity [18]. P1B-ATPases play important roles in the transport and homeostasis of metals in plants [19]. The P1B-ATPase subfamily has eight members in *Arabidopsis thaliana* [20]. In previous reports, *AtHMA1* to *AtHMA4* transport divalent cations, whereas *AtHMA5* to *AtHMA8* transport monovalent cations [19,21]. These genes play a primary and important role in metal translocation from the root to the shoot and subsequent metal accumulation within the shoot [22]. *AtHMA3* was verified to play an important role in Cd tolerance and accumulation in shoots and is also able to transport other metals, including Zn, Co, and Pb [23]. In rice, *OsHMA3* is mainly expressed in rice roots and its expression is nonresponsive to Cd exposure. *OsHMA3* knockout resulted in increased Cd translocation from roots to shoots, whereas the overexpression of *OsHMA3* had the opposite effect [24,25]. *OsHMA4* and *OsHMA5* are shown to be involved in loading Cu to the xylem for root-to-shoot translocation [26].

To cope with high levels of Cd stress, the first point of defense for a plant is its cell wall in the root system, which prevents Cd from entering the cell [27]. Under Cd stress, maize seedlings enhance their tolerance and defend against Cd by increasing the activity of antioxidant enzymes in plant tissues. When maize seedlings were cultivated in a Cd solution, the proportion of the root cell wall area and content of b-1,3-glucan subtypes, compared with the control, had significantly increased, which may be attributed to the response of their Cd tolerance mechanism [28]. Herein, improving the Cd tolerance and reducing the grain Cd content play important roles in increasing maize yield and quality. The members of the HMA family, an important group of heavy metal transport proteins, were proven to be involved in Cd transport in other plants, but few studies have investigated these proteins in relation to maize. In this study, we demonstrate that the maize HMA family member, ZmHMA3, is located on the cell membrane. The mutant of the homologous gene *AtHMA2* showed Cd sensitivity in *Arabidopsis*. The overexpressed transgenic plants were described from maize *ZmHMA3* to prove their role in Cd and Zn absorption and transport. Therefore, this study is expected to provide a theoretical basis for improving maize’s Cd and Zn stress tolerance in the future and reducing the risks of heavy metal contamination in maize cultivation through biotechnological tools.

## 2. Results

### 2.1. Phylogenetic Tree and the Conserved Structural Domain Analysis of Maize HMA Family Genes

**Figure 1:**
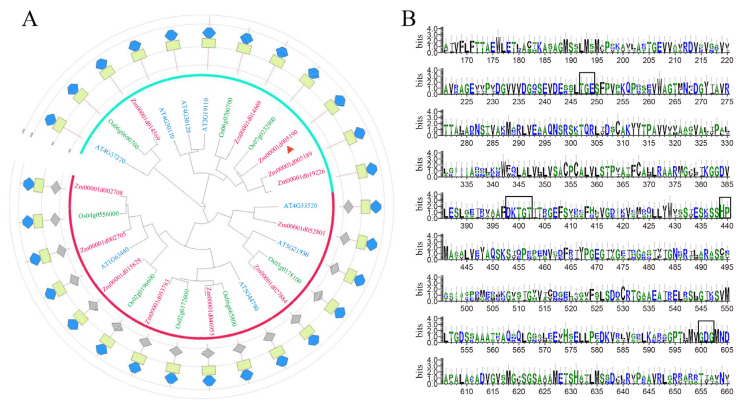
Phylogenetic construction and conservative sequence analysis of HMA family genes: (**A**) phylogenetic analysis of the HMA family in maize, rice, and *Arabidopsis*; (**B**) conservative sequence analysis of ZmHMA3 and OsHMA2, 3 in rice. Note: (**A**), the pink font represents the ZmHMA family, the green font represents the OsHMA family, and the blue font represents the AtHMA family. The red triangle refers to ZmHMA3; (**B**), the height of individual amino acids in the stack shows the relative frequency of such amino acids in this position, and the amino acid residues within black boxes are conserved functional domains in the HMA family.

To understand HMA family genes, we obtained the species libraries of maize, rice, and *Arabidopsis* (accessed on 5 January 2018, https://plants.ensembl.org/index.html) by using the conserved structure of the HMA family (P-type ATPase, subfamily IB). The amino acid sequences of ZmHMA3, OsHMA2, OsHMA3, AtHMA2, and AtHMA3 were accessed by the gramene website. Multiple sequence alignments were performed, which show that the amino acid sequence of ZmHMA3 is extremely conserved with that of HMA in other species (Figure S1). We found that the ZmHMA family had 12 members in maize, 8 members in rice, and 8 members in *Arabidopsis,* by using the online website (accessed on 5 January 2018, clustalo: https://www.ebi.ac.uk/Tools/msa/clustalo/). The sequences of *ZmHMA*, *OsHMA,* and *AtHMA* were analyzed via multiple sequence alignment, and the phylogenetic tree was constructed using MEGA6. The results showed that *ZmHMA3* (*Zm00001d005190*) was most closely related to the *OsHMA3* gene (*Os07g0232900*), and the conserved structural domains of the HMA family were predicted using interpro/Searc (accessed on 5 January 2018, Pfam: https://www.ebi.ac.uk/Tools/pfa/pfamscan/) (Figure 1A). The HMA family contains E1-E2 ATPase structural domains, and hydrolase structural domains (haloacid dehalogenase-like hydrolase). The height of individual amino acids in the stack shows the relative frequency of such amino acids in this position, and those marked with black boxes represent amino acid residues that are conserved in three important functional regions of the HMA family, such as TGE, a phosphatase domain located in the functional region of the HMA family. The DKTGT, a histidine kinase domain, is involved in the energy transduction. HP is located in the P functional region of HMA with an ion conversion motif. GDG represents an ATP-binding domain, which is located in the N-functional region of HMA (Figure 1B).

### 2.2. Functional Expression of ZmHMA3 in Response to Cd Stress

**Figure 2:**
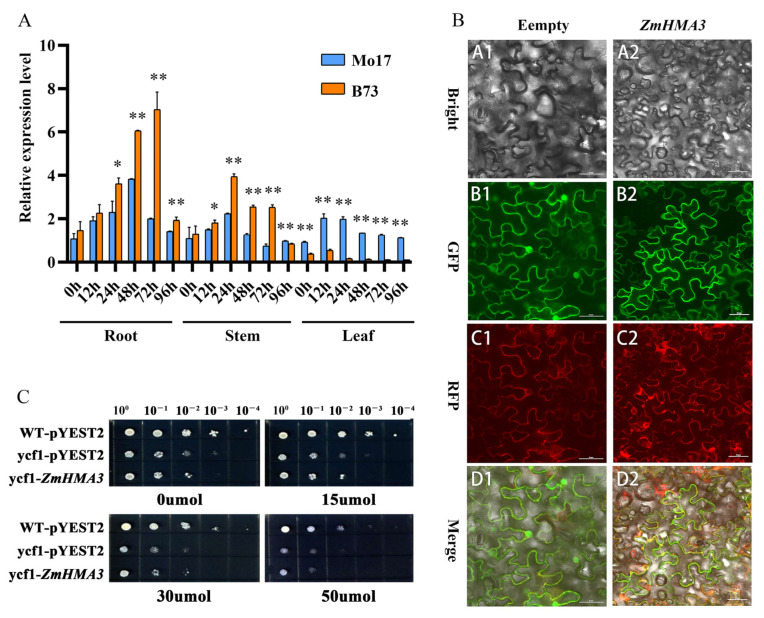
Expression pattern analysis of *ZmHMA3* in different tissues, subcellular localization of *ZmHMA3* in tobacco leaves, and heterologous expression analysis of the *ZmHMA3* in yeast cells: (**A**) analysis of expression patterns of *ZmHMA3* in different tissues; (**B**) localization observation of *ZmHMA3* in tobacco leaves; (**C**) heterologous expression stress of *ZmHMA3* in yeasts under stress; * means *p* ≤ 0.05; ** means *p* ≤ 0.01; Student’s *t*-test. Note: (**A**), the figure shows the relative expression of the *ZmHMA3* gene in roots, stems, and leaves of maize B73 after 12, 24, 48, 72, and 96 h of Cd stress at 200 μmol/L concentration; (**B**), the GFP server indicates the green fluorescent protein, the RFP server indicates the red fluorescent protein, the bright color indicates the cell morphology image, and the merged pattern indicates a combined image of dark filed fluorescence and cell morphology images. The scale bar is 30 μm; (**C**), the *X*-axis direction shows the 10 gradients of the diluted yeast liquid from left to right, and the *Y*-axis direction indicates the transformation of yeast strains with different target vectors.

The RNAs of the roots, stems, and leaves of maize varieties B73 and Mo17 were obtained at different treatment times under a Cd concentration of 200 μmol/L stress. The expression pattern of the *ZmHMA3* gene was analyzed using qRT-PCR, and the plant with no Cd stress was used as the control. As shown in Figure 2A, the expression of *ZmHMA3* was higher in B73 tissues than in Mo17 tissues of roots and stems under Cd stress treatment (Figure 2A). After 72 h of Cd stress treatment, the expression of *ZmHMA3* in the root was highest compared with other times. Moreover, the expression of *ZmHMA3* in B73 was highly and significantly different compared with Mo17, which was over four times higher than the control. In the stems, the *ZmHMA3* expression reached the highest at 24 h and then decreased. The *ZmHMA3* expression in B73 was significantly higher than Mo17 between 24 h and 72 h. Interestingly, the expression of *ZmHMA3* was significantly higher in the leaf of Mo17 compared with that of B73. These results indicate that *ZmHMA3* is mainly expressed in roots under Cd stress, and B73 has a better ability to resist Cd stress.

### 2.3. Subcellular Localization of ZmHMA3 and Cd Sensibility in Yeast

To study the function of *ZmHMA3*, we next investigated the subcellular localization of *ZmHMA3*, for which the recombinant plasmid and empty vector were transferred into the lower epidermal cells of the leaves of Ben’s tobacco. After 48 h of expression, it was observed via fluorescence confocal microscopy. The results revealed that *ZmHMA3* was mainly expressed on the cytoplasmic membrane (Figure 2B). Thus, we inferred that the ZmHMA3 protein was localized on the cell membrane.

To study the influence of *ZmHMA3* on yeast tolerance, *ZmHMA3* was constructed into the pYES2 vector and transformed into the yeast mutant strain Δycf1. Under the stress induced by 0 μmol/L and 15 μmol/L Cd concentrations, WT, Δycf1, and Δycf1-*ZmHMA3* showed no difference (Figure 2C). However, Δycf1-*ZmHMA3* was more sensitive and grew worse than WT and Δycf1 on a 30 μmol/L Cd-containing medium. The same results were found for a 50 μmol/L medium (Figure 2C). Thus, *ZmHMA3* transformation increased the sensitivity of yeast to Cd stress in the yeast mutant strain. These results implied that *ZmHMA3* may have a role in Cd transport from outside to inside in the yeast cell, causing the mutant strain to become more sensitive under high Cd stress.

### 2.4. Mutant of AtHMA2 Decreased Resistance to Cd Stress in Arabidopsis

**Figure 3:**
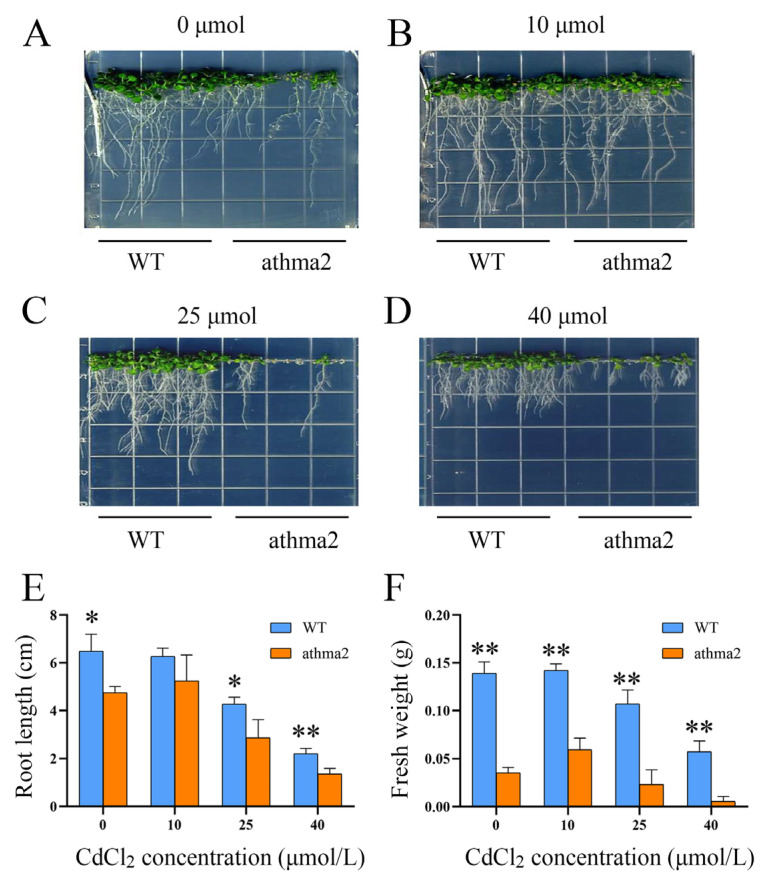
Phenotype identification and root trait analysis of *AtHMA2* homozygous in mutants: (**A**–**D**) phenotypic identification of *Arabidopsis AtHMA2* homozygous mutants under different concentrations of Cd stress; (**E**,**F**) statistical plots of root length and fresh weight were measured under Cd stress; * means *p* ≤ 0.05; ** means *p* ≤ 0.01; Student’s *t*-test.

To study the function of the HMA family under Cd stress, we obtained a homozygous mutant of *Arabidopsis* to conduct Cd stress experiments. Four CdCl_2_ stress concentrations were set at 0 μmol/L, 10 μmol/L, 25 μmol/L, and 40 μmol/L. The results showed that, under 0 μmol/L conditions, the mutant of *athma2* led to weaker growth compared with WT. However, the mutants restored growth under 10 μmol/L CdCl_2_ stress (Figure 3A,B). Herein, the growth of *Arabidopsis* was promoted using the low concentrations of heavy metals, whereas the root length of *athma2* was significantly decreased compared with WT at the Cd concentration of 25 μmol/L and 40 μmol/L (Figure 3C,D). The measurement statistics of their root length and fresh weight showed the same results, as shown in Figure 3E,F. These results suggest that the *AtHMA2* gene is involved in Cd tolerance.

### 2.5. Overexpression of ZmHMA3 Increased Tolerance in Heavy Metal Stress

**Figure 4:**
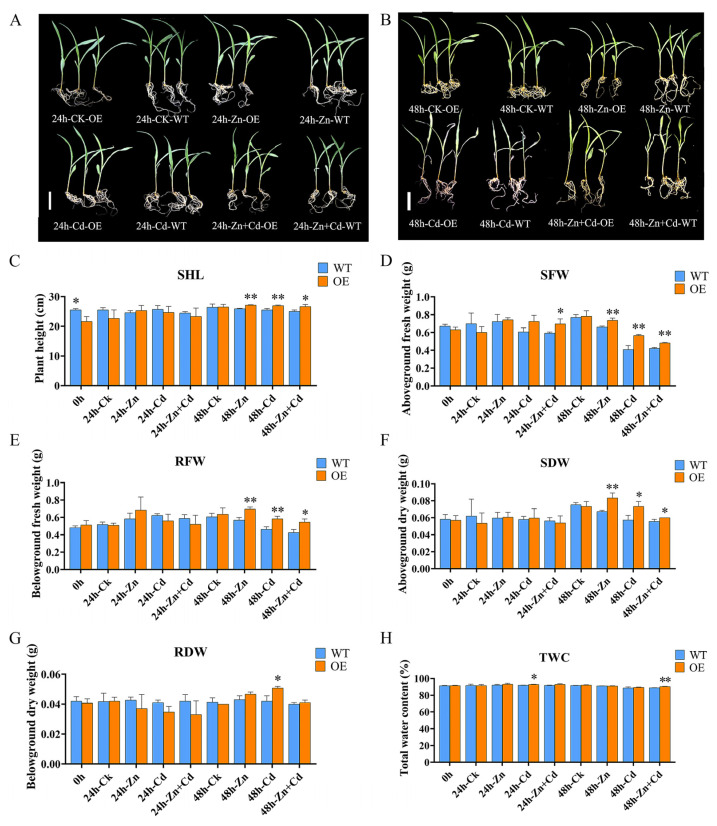
Comparison of agronomic traits between overexpressed transgenic *ZmHMA3* and wild-type plants at different times and under different treatments: (**A**) the seedling grown under 24 h of heavy metal treatment; (**B**) the seedling grown under 48 h of heavy metal treatment; the scale bar is 5 cm; (**C**) seedling plant height (SHL); (**D**) seedling fresh weight (SFW); (**E**) root fresh weight (RFW); (**F**) seedling dry weight (SDW); (**G**) root dry weight (RDW); (**H**) total water content; * means *p* ≤ 0.05; ** means *p* ≤ 0.01; Student’s *t*-test.

To identify the *ZmHMA3* function of Cd stress, we investigated the overexpression (OE) of transgenic maize, and its relative expression is shown in Supplement Figure S2. We compared the relative expression and agronomic traits of maize seedlings under different levels of heavy metal stress (Figure 4). The B104 (WT) and OE plants were treated with 800 μmol/L CdCl_2_·2.5H_2_O, 800 μmol/L ZnSO_4_·7H_2_O, and 800 μmol/L CdCl_2_·2.5H_2_O + 800 μmol/L ZnSO_4_·7H_2_O solutions for a certain period of time. A comparison of the growth phenotype between B104 and OE plants was shown in Figure 4A,B. Compared with CK under 24 h Cd stress treatment and 24 h Zn stress treatment, no significant differences in plant heights were observed. Under 24 h Zn + Cd stress treatment, the first leaf of the WT plant wilted, and OE plants grew better than the wild type (Figure 4A). Under 48 h Cd stress treatment, 48 h Zn stress treatment, and Zn + Cd stress treatment, WT and OE plants underwent wilting, and the degree of wilting was higher in WT plants than in OE plants (Figure 4B).

Moreover, we measured a set of physiological indices in WT and OE plants, including the seedling plant height (SHL), the seedling fresh weight (SFW), the root fresh weight (RFW), the seedling dry weight (SDW), the root dry weight (RDW), and the total water content (TWC) (Figure 4C–H). The results showed no significant differences were observed between OE and WT in any of these traits before 48 h, except for SFW and TWC in 24 h heavy metals stress treatment. However, most OE plants showed obvious significant differences in these traits compared with WT under 48 h Zn stress. The above-ground fresh and dry weights of wild-type plants were generally less than those of OE plants under different stress conditions and time periods. Under 48 h of Cd stress treatment, the measured traits were superior in OE plants compared with wild-type plants. These results indicate that OE-*ZmHMA3* increases tolerance under heavy metal stress.

### 2.6. The Effects of Heavy Metal Stress on Maize: Root System Responses, Cd and Zn Accumulation and Translocation, and Subcellular Cd and Zn Distribution

**Figure 5:**
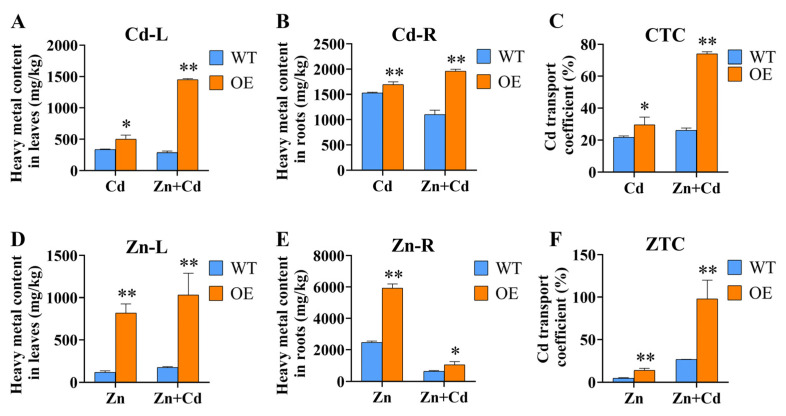
Zn content, Cd content, and transport coefficients determined in *ZmHMA3*-overexpressed and wild-type plants under hydroponic conditions: (**A**) above-ground Cd content; (**B**) below-ground Cd content; (**C**) Cd transport coefficient; (**D**) above-ground Zn content; (**E**) below-ground Zn content; (**F**) Zn transport coefficient; * means *p* ≤ 0.05; ** means *p* ≤ 0.01; Student’s *t*-test.

The plant’s root system is the first tissue that comes into contact with heavy metals, and some heavy metals are absorbed into root cells and transferred to the parts of the plant above ground. The accumulation of Cd in the root system may be a way for plants to cope with Cd stress [29]. We detected heavy metal content in WT and OE plants under different stress treatments. The results showed that OE plants accumulated higher heavy metal content than WT under Cd, Zn, and Cd + Zn stress (Figure 5). Under Cd stress treatment, all OE plants had higher Cd content than WT, and the Cd transport coefficient was significantly different from that of WT (Figure 5A–C). Regarding the Zn content, the Zn content of OE plants was obviously significantly higher than WT in leaves (Figure 5D). Moreover, the heavy metal content of OE plants was obviously less under Cd + Zn stress than under Zn stress (Figure 5E). The same trend was observed with the Zn transport coefficient and the Cd transport coefficient (Figure 5F). These results suggest that *ZmHMA3* might play an important role in heavy metal transport.

**Figure 6:**
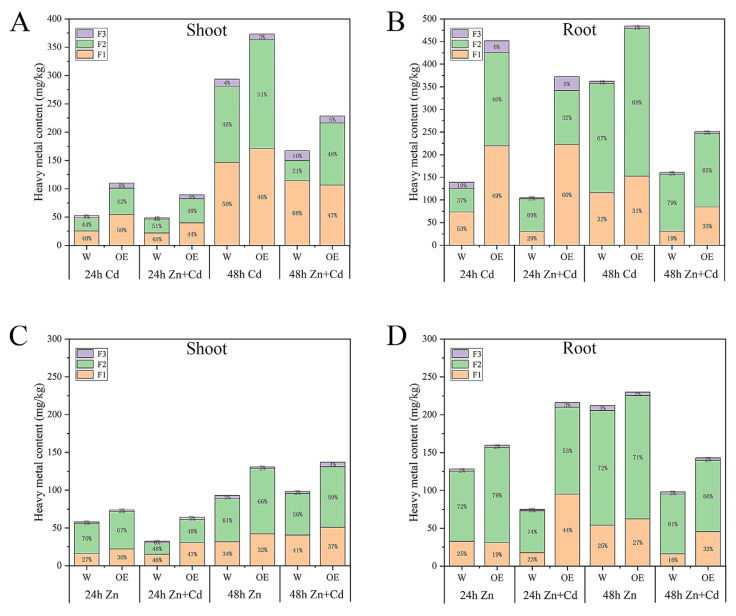
The accumulation of heavy metals in *ZmHMA3*-overexpressed and wild-type plants was investigated in above- and below-ground parts of the plants. Subcellular distribution in Cd treatment: (**A**) shoots; (**B**) roots. Subcellular distribution in Zn treatment: (**C**) shoots; (**D**) roots.

To understand the accumulation of heavy metal content in different tissues, we determined the Cd and Zn contents of cell walls (F1), cytoplasm and vacuole (F2), and membranes and organelles (F3) in WT and OE plants. Under Cd stress, the Cd concentration in the root was higher than that in the shoot under almost all treatments and time points. The different organelles of OE had higher Cd concentrations than WT. Under Zn + Cd stress, the OE plants had the same traits as plants treated with Cd concentration in different organelles, but Cd accumulation was less under Zn + Cd stress treatment than under Cd treatment alone. These results indicate that Cd was highly mobile in the OE plants (Figure 6A,B). Furthermore, the F1, F2, and F3 had higher Zn concentrations in roots than in shoots in the OE plants (Figure 6C,D). In all treatments and time points, the OE plants showed higher Cd concentrations than WT, especially in F1 and F2. Additionally, the OE plants treated with Zn + Cd for 24 h had lower levels of heavy metals than plants treated with Zn alone. These results indicated that OE plants had a strong ability to transfer heavy metals. However, considering OE and WT plants treated with Zn + Cd for 48 h, it was found that heavy metal content in all organelles markedly decreased. This phenomenon might be due to plant injury.

## 3. Discussion

Most HMA proteins are involved in essential and nonessential metal transportation, including Cd and Zn. A previous study revealed that the *ZmHMA3* protein, a P-type ATPase heavy metal transporter, can regulate Cd accumulation in maize seeds [30]. The *ZmHMA3* gene was identified through linkage disequilibrium analysis and QTL colocalization under Cd stress, and its expression was quantified in different tissues under Cd stress [31]. However, the function of *ZmHMA3* is not clear under Cd and Zn stress. Structural analysis showed that ZmHMA3 belongs to the Zn/Cd subclass and contains eight transmembrane structural domains with conserved E1-E2 ATPase domains. ZmHMA3 is homologous with HMA2 and HMA3 in rice and *Arabidopsis* (Figure 1A and Figure S1). ZmHMA3 will sensitively disappear since the conserved aspartic acid phosphorylation site has mutated into alanine. In addition, ZmHMA3 contains eight transmembrane domains, which is the P functional domain of phosphorylation sites and MBD metal-binding sites in the conserved motifs [30,32]. The phosphorylation site’s P functional domain was found on the cytoplasmic side of the transmembrane structure of ZmHMA3, which was involved in the transmembrane transport of heavy metals (Figure 1B).

Subcellular localization analysis showed that *ZmHMA3* was localized in the cell membrane of tobacco leaves. Quantitative analysis revealed that *ZmHMA3* had higher expression in B73 roots than in Mo17 (Figure 2A). *ZmHMA3* was upregulated in roots and stems under Cd stress, and this result is similar to previous studies on *OsHMA2*, *AtHMA2*, and *HvHMA2* [33]. These genes are involved in transporting metals from the subsurface to the above ground, and they have various functions in transporting metals into or out of cells and in loading or unloading metals in the xylem [33,34].

According to the mechanism of action of *HvHMA2* in barley, after *HMA2* is transformed into yeast strains, it is expressed in the yeast’s extracellular membrane and some intracellular membrane systems. An investigation of the heterologous expression of *HvHMA2* in yeast demonstrated that it was expressed on the yeast PM and ER membranes. The ER membrane system is sensitive to heavy metals, and transgenic-positive strains were found to be more sensitive to Cd than WT [15,35]. The *ZmHMA3* transformation strain of yeast was expressed under Cd stress, which showed higher sensitivity than the empty vector (Figure 2C). This result was similar to the results of *ZmNRAMP2* and *HvHMA2* in a previous report [31]. The heterologous expressions of *OsHMA2* and *OsHMA3* were investigated to explore Cd tolerance in yeast, and it was found that *OsHMA3* was expressed on the vacuolar membrane. Thus, the different structures of proteins in the HMA family lead to functional differences in plants [1,36].

The *Arabidopsis HMA2* homozygous mutant was utilized to confirm the sensitivity of *AtHMA2* under Cd stress. The expression of *AtHMA2* was found to be concentrated on the cell membrane in the vascular bundle, which is responsible for transporting metal ions from the roots to the above-ground parts. In the homozygous mutant plants, which were deficient in Cd ion transport, Cd was accumulated in the roots, leading to inhibited growth [37]. Our results showed that the *athma2* mutant led to weak growth under Cd stress (Figure 3). Upon stress treatment of overexpressed plants, it was observed that their growth status was better than that of the wild type [38,39]. The addition of Zn affected the uptake of Cd in the plants, and this result was consistent with previous studies. Agronomic traits were measured, showing that the above-ground fresh weight and the below-ground dry weight of the wild type were lower than those of OE plants under 48 h treatment with different stress conditions (Figure 4).

Overall, the overexpressed plants showed better growth than the wild type in terms of root phenotypes (Figure 4E,G,I). This result indicates that OE-*ZmHMA3* can enhance the ability to translocate and efflux Cd and Zn. The distribution of Cd and Zn content in different subcells revealed that *ZmHMA3* translocated Cd and Zn into the cytoplasm and alleviated Cd and Zn damage to the cell membrane (Figure 6). Cd concentration in both OE and WT plants increased with stress duration under Cd treatment, while Cd concentration decreased in OE-*ZmHMA3* plants under Zn + Cd treatment. This phenomenon was mainly due to the high content of heavy metals, resulting in absorption disorders (Figure 6A,B). These results are consistent with those of previous studies [40]. These findings enhance our understanding of the function of *ZmHMA3* in improving maize growth and resistance to Cd and Zn stress. This study provides a reference for improving the characteristics of heavy metal enrichment in existing maize varieties and the plant remediation technology of heavy-metal-contaminated soil.

## 4. Materials and Methods

### 4.1. Plant Materials and Growth Conditions

The self-incompatible maize lines B73, B104, and Mo17 were provided by Sichuan Agricultural University, and Colombian wild-type *Arabidopsis* and the *AtHMA* mutant were purchased at ABRC. The transient expression receptor Ben’s tobacco was provided by Mr. Li Ping’s group at the Institute of Rice Research, Sichuan Agricultural University. The *Arabidopsis* plants were grown in a short daylight incubation chamber for 2 months, and then imbibed seeds were harvested and cultured in a 1/2 MS solid medium supplemented with CdCl_2_. Ben’s tobacco was planted in pots for 3 or 4 weeks with light incubation, and the plants were used to conduct a subcellular localization assay.

### 4.2. ZmHMA3 Expression Analysis Using Real-Time Quantitative PCR

*ZmHMA3* expression patterns were determined using an ABI 7500 real-time PCR system (Torrance, CA, USA). B73 maize seedlings were treated with 200 μmol/L CdCl_2_ for 12 h, 24 h, 48 h, 72 h, and 96 h. TRIzol reagent (Invitrogen, Gaithersburg, MD, USA) was used to extract total RNA from roots, stems, and leaves of B73 maize seedlings, and first-strand cDNA was synthesized using the primeScript^TM^RT 1st Strand cDNA Synthesis kit (Code Ds10A, TaKaRa, Kyoto, Japan). The synthesized cDNA was amplified using 200 nM of gene-specific primers and 10 μL of SYBR Green SuperMix (Bio-Rad, Santa Clara, CA, USA). The amplification program was set as follows: 95 °C for 3 min, 95 °C for 15 s, 60 °C for 1 min, 40 cycles, and 65 °C to 95 °C to generate a melting curve with a duration of 1 s. GAPDH was used as an internal reference gene, and three technical replicates were designed for each sample. The relative expression level of *ZmHMA3* under different treatment conditions was analyzed using the 2^−ΔΔCT^ method. All primers used in this study are listed in Table S2.

### 4.3. Subcellular Localization Assay

The *ZmHMA3* of B73 was cloned and conducted into the pCAMBIA2300-35S-eGFP vector, and positive expression vectors were transformed into GV3101 agrobacterium cells. The empty vector was used as the control and treated similarly. The resuspended cells were injected into tobacco plants using a syringe with a resuspension solution containing 10 mmol/L MgCl_2_ and 200 µmol/L MES (2-(4-Morpholino) ethanesulfonic acid). The tobacco plants were cultured under normal conditions for 36–72 h, and the expression site of the gene was observed under a confocal microscope. Marker prediction and recombination vector colocalization were carried out to determine the expression site of the gene.

### 4.4. Yeast Heterologous Expression

The WT (BY4741) yeast strain was used in this study, which is a Cd-sensitive mutant yeast strain Δycf1. The full-length CDS of *ZmHMA3* was cloned from the B73 line into the pYES2 vector driven by the GAL1 promoter to produce the recombinant construction pGAL1: *ZmHMA3*. The yeast mutant strain Δycf1 was transformed using the PEG method, and after diluting 4 gradients of a yeast solution in 10 gradients, 3 μL drops of yeast solution from 5 gradients were pipetted onto SC-U galactose solid induction medium containing 0 μmol/L, 15 μmol/L, 30 μmol/L, and 50 μmol/L and incubated at 30 °C for 2–3 days. The Δycf1 strain was the negative control, and the WT strain BY4741 was the positive control. The growth of WT, the transfer of the mutant strains to null, and the transformation of the yeast mutant strain with *ZmHMA3* were assessed.

### 4.5. Genetic Transformation of Arabidopsis Thaliana

T-DNA-inserted *Arabidopsis* homozygous mutant seeds were purchased from the ABRC company. The mutant seeds were collected and subjected to stress verification. The T_1_ generation seeds were screened under different Cd stress treatments. Briefly, the 1/2 MS solid medium was the basis medium, and different Cd concentrations were added to this medium, namely 0, 25, 40, and 50 µmol/L. Then, 10 seeds were inoculated per dish for each type of treatment, with 3 replicates. Root length, root hair growth, and above-ground growth were analyzed.

### 4.6. Overexpression of ZmHMA3 in Maize

The amino acid sequences were translated by the online translation software ExPASy (accessed on 20 January 2018, https://web.expasy.org/translate/). The CDS region of *ZmHMA3* was constructed into a CUB overexpression vector. The CUB-*ZmHMA3* fusion protein was transformed into young maize embryos via the Agrobacterium-mediated imbibition of young maize embryos. The positively overexpressed transgenic plants were examined using specific primers with the vector *bar* gene. B104 was used as the negative control. A total of 17 transgenic plants were harvested from the T_0_-generation-positive seeds. The transgenic plants were developed for T_2_ generation via self-fertilization. The seeds with positive overexpression of *ZmHMA3* were detected with the endosperm DNA.

### 4.7. Treatment of Cd and Zn Stress in Maize Seedlings

All of the seeds were treated with deionized water in the experiment, soaked in 20% H_2_O_2_ washed for 20 min, and then washed with deionized water 4–6 times for 3 min each time. Finally, the seeds were germinated in an artificial climate chamber on germination paper (Anchor paper, St. Paul, MN, USA), which was rolled up and placed in a small bucket filled with double-distilled water for germination [41]. When the seedlings reached the stage of two old leaves and one new leaf, the endosperm was removed and divided into four groups. The first group was the control, the second group was treated with Zn (800 μmol/L ZnSO_4_·7H_2_O), the third group was treated with Cd (800 μmol/L CdCl_2_·2.5H_2_O), and the fourth group was treated with Zn + Cd (800 μmol/L ZnSO_4_·7H_2_O + 800 μmol/L CdCl_2_·2.5 H_2_O). These materials were transferred to a semi-nutrient culture for 2 days, after which it was changed to a full-nutrient culture (Table S1). Moreover, oxygenation was monitored throughout culture development and the simultaneous stress treatment, and the maize plants were observed after 0 h, 24 h, and 48 h to analyze the phenotype and determine the physiological and biochemical indices of the plants treated with different materials. All experiments were performed with three biological replicates.

### 4.8. Determination of Agronomic Traits and Physiological Indicators in Maize Seedlings

Maize seedlings were used under the above four different treatments and three different time periods with biological replicates of 20. The agronomic traits were analyzed including the height, above-ground fresh weight, and below-ground fresh weight at different stress times. To determine the dry weight, the roots and above-ground parts were dried in an oven at 85 °C. Three biological replicates were set up by taking the root systems of hydroponically grown seedlings under treatment with different materials for 24 h and 48 h.

### 4.9. Uptake and Accumulation of Cd and Zn in Maize Seedlings

(1) Determination of Cd or Zn content

Sample processing: The samples with 0.2 g dry weight were placed in a digestion tube. Then, 10 mL of nitric acid (AR) was added to a microwave digestion device for 1 h, and the acid was dried at 180 °C for 70 min until the remaining liquid in the digestion tube was about 0.5–1 mL. After cooling to room temperature, the sample was filtered into a 10 mL volumetric flask, and the volume was increased by washing with deionized water. For the configuration of the standard curve, 100 μg/mL of Cd^2+^ and Zn^2+^ standard solution was used as the master solution and diluted into 10 concentration gradients of 5 ng/mL, 10 ng/mL, 15 ng/mL, 20 ng/mL, 30 ng/mL, 50 ng/mL, 60 ng/mL, 70 ng/mL, 80 ng/mL, and 100 ng/mL to make the standard curve. For content determination, an experiment was carried out using plasma emission spectrometry with a full-spectrum instrument (iCAP5110). The Cd^2+^ and Zn^2+^ standards were first determined, and the standard curves were plotted with linear correlation coefficients R^2^ > 0.999. Then, the Cd and Zn contents in the samples were determined, and three biological replicates were performed. Formula:

*X* = (*C × V × f ×* 10^−6^)/(*M ×* 10^−3^)

where *X* represents the content of Cd or Zn in the sample in mg/kg; *C* represents the Cd concentration of the solution to be measured in ng/mL; *V* represents the fixed volume of the solution to be measured in mL; *f* is the dilution multiple of the solution to be measured; and *M* represents the mass of the sample in g.

(2) Determination of different Cd and Zn concentrations in the subcellular analysis

A differential centrifugation method was used to determine the Cd and Zn contents in the subcellular analysis of maize seedlings under different treatments at different time periods. Briefly, 1 g of each fresh sample was accurately weighed in a 10 mL centrifuge tube, and 10 mL of extraction buffer (250 mmol/L sucrose, 50 mmol/L Tris-HCl (pH 7.5), and 1 mmol/L dithiothreitol) was added. The cell wall fraction (noted as F1) was precipitated through rapid grinding into a homogenate in a mortar under ice-bath conditions and centrifuged at 3000 rpm/min for 15 min; the supernatant was centrifuged at 11,000 rpm/min for 45 min, and the precipitate was the membrane and organelle fraction (noted as F3) and the supernatant was the soluble fraction/cytosol and cytoplasmic fraction (noted as F2). The content was measured via sterilization, and the volume was determined in the same way as above (1). The entire operation was carried out at 4 °C [42,43].

### 4.10. Statistical Analysis

All the data were analyzed using BM SPSS Statistics 26, GraphPad Prism 8, and Excel 2016, and significant differences among samples were compared using Student’s *t*-test.

## 5. Conclusions

In this study, we investigated *ZmHMA3,* which is a regulator of Cd and Zn transport absorption in maize. *ZmHMA3* encodes an HMA family protein, which belongs to the subfamilies of P-type ATPase. The ZmHMA3 protein was localized on the cell membrane of tobacco, and the analysis of yeast tolerance showed that *ZmHMA3* may play a role in transporting Cd from outside to inside in the yeast cell. The homologous mutants of *AtHMA2* showed Cd sensitivity compared with WT. *ZmHMA3*-overexpressed plants revealed healthier growth under different Cd and Zn stress, and subcellular distribution analysis showed that OE-*ZmHMA3* plants had higher Cd and Zn accumulation in different tissues. These findings enhance our understanding of the *ZmHMA3* function and genetic mechanisms, and this study proposes a potential method to improve soil contaminated by heavy metals.

## Data Availability

The original data presented in the study are included in the article/Supplementary Materials. Further inquiries can be directed to the corresponding author(s).

## Supplementary Materials

The following supporting information can be downloaded at: https://www.mdpi.com/article/10.3390/ijms241713496/s1.
