# Supplementary material for: ZmHMA3, a Member of the Heavy-Metal-Transporting ATPase Family, Regulates Cd and Zn Tolerance in Maize
Source: Int J Mol Sci. 2023 Aug 30;24(17):13496. doi: 10.3390/ijms241713496 (PMC10487686; doi:10.3390/ijms241713496)
Supplement: Supplementary file 1 [file ijms-24-13496-s001.zip › ijms-2538555-supplementary.pdf]

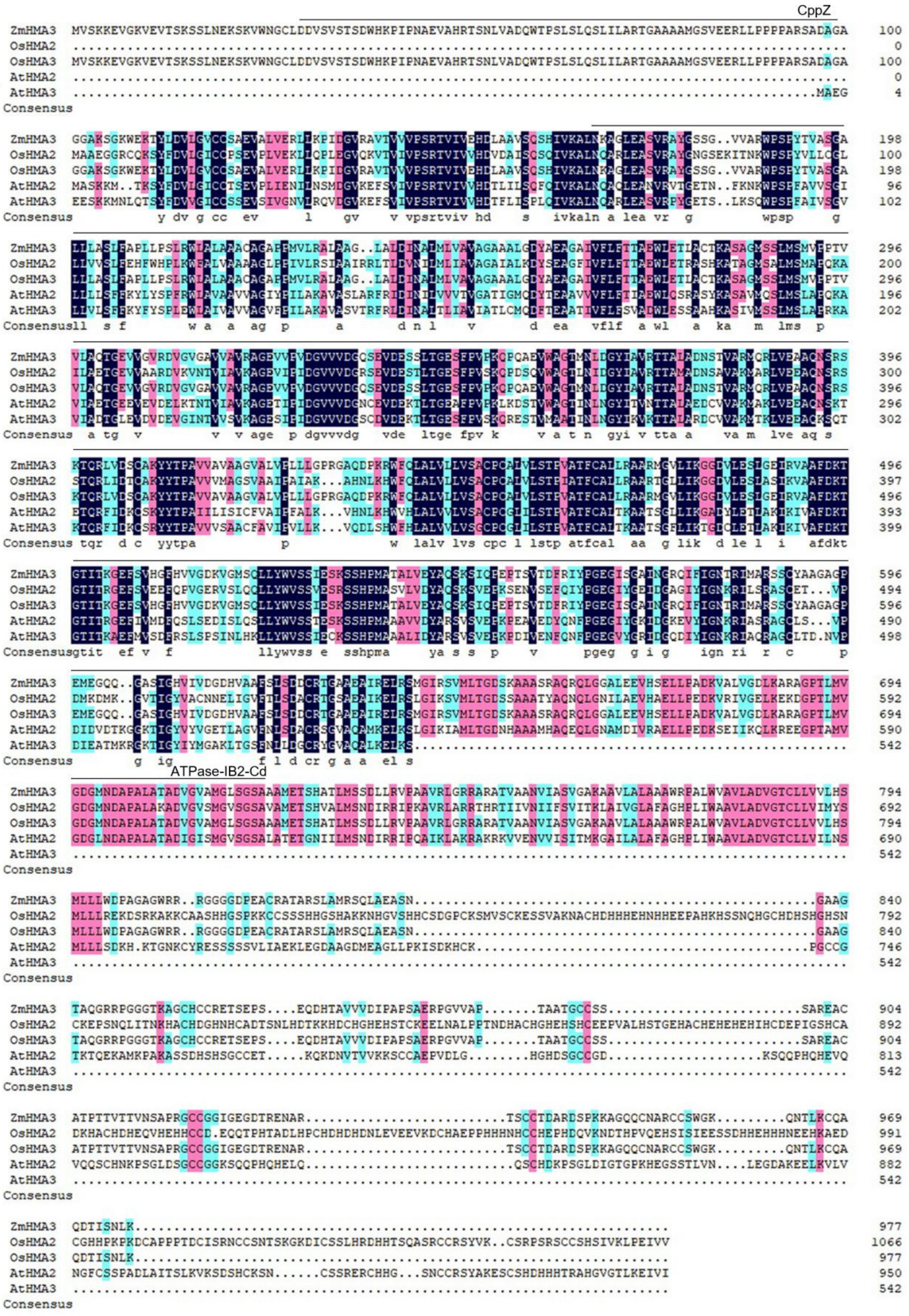

Figure S1. Multi-sequence alignment of ZmHMA3, OsHMA2, OsHMA3, AtHMA2,, and AtHMA3 amino acids using DNAman software.

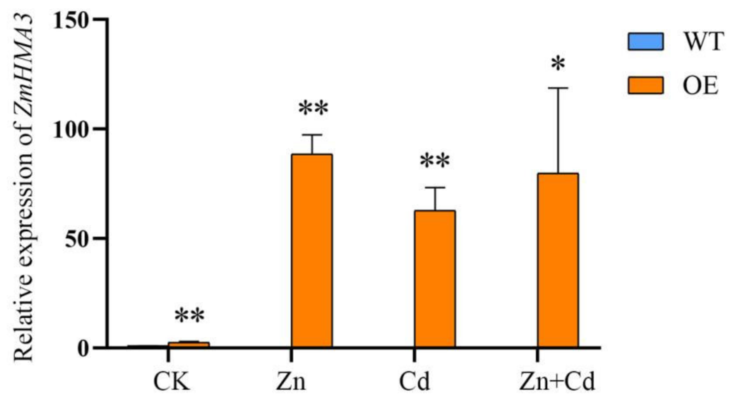

Figure S2. Relative expression of *ZmHMA3* in WT and overexpressed transgenic plants. Note: CK represents treatment with no heavy metal solution, Zn represents treatment with 800 μmol/L solution of $ZnSO_4$, Cd represents treatment with 800 μmol/L solution of $CdCl_2$, and Zn+Cd represents treatment with 800 μmol/L solution of $ZnSO_4$ +$CdCl_2$; * means $P \leq 0.05$;** means $P \leq 0.01$; Student's *t*-test.

Table S1. Hydroponic nutrient solution formula for maize seedlings.

Drug Name		Molar mass	Mass
Ca(NO3)2·4H2O	4 mmol/L	236.5	95 mg
Fe·EDTA	10 μmol/L	367.47	36.747 mg
KH2PO4	1 mol/ L	115.03	115.03 g
(NH4)6Mo7O24(4H2O)	0.016 μmol/L	1235.6	0.198 g
MnCl2·4H2O	0.1 μMol/L	197.91	1.81 mg
MgSO4·7H2O	2 mmol/L	246.47	492.3 mg
KNO3	6 mmol/L	101.1	606.6 mg
NH_4NO_3	1 mmol/ L	80	80 mg
$CuSO_4$·$7H_2O$	0.1 μmol/L	249.69	0.0249 mg
H_3BO_3	1 μmol/L	61.83	62 μg
$ZnSO_4$·$7H_2O$	1 μmol/L	132	132 μg

Table 2. All primers in experiment.

Name	Premier sequence Forward (5'---3')	Premier sequence Reverse (5'---3')	Used
ZmHMA3-GFP	CTTGGTACCGAGCTCGGATCCATGGGCAGTGTCGAGGAGAG	GCGGCCGTTACTAGTGGATCCTCACTTGAGATTTGAGATTGTA	Subcellular localization
ZmHMA3	ATGGTTTCAAAGAAGGAAGTGGGC	TCACTTGAGATTTGAGATTGTATC	Clone
q-ZmHMA3	ATGTGGTTGGGGACAAGGTTGG	ATCTCAGCTCACGGATCGCCTCT	RT-PCR
Yeast-HMA3	CGACTCTAGAGGATCCATGGGCAGTGTCGAGGAGAG	CGGTACCCGGGGATCCTCACTTGA GATTTGAGATTGTA	Yeast Heterologous Expression
OE-ZmHMA3	CGGGGGTACCGGATCCATGGGCAGTGTCGAGGAGAG	TTCAGAATTCGGATCCTCACTTGAGATTTGAGATTGTA	Over-expression
Bar-gene	GCCATCTATGTTCTCCTGTTGACT	ATCTTGCGTGGAGGTGTGTG	Transgenic detection
GAPDH	CCATCACTGCCACACAGAAAAC	AGGAACACGGAAGGACATACCAG	Reference gene
